# Evaluation of Reusable Thermal Protection System Materials Using a High-Velocity Oxygen Fuel Torch

**DOI:** 10.3390/ma17215229

**Published:** 2024-10-27

**Authors:** Rajesh Kumar Chinnaraj, Minjeong Kim, Bogyu Choi, Taerin Ha, Seongwon Kim, Min-Soo Nam, Seong Man Choi

**Affiliations:** 1 Department of Aerospace Engineering, Jeonbuk National University, Jeonju-si 54896, Republic of Korea; rajkumchin@jbnu.ac.kr (R.K.C.); gmj1711@jbnu.ac.kr (M.K.); cjyhero@jbnu.ac.kr (B.C.); xoflsgk@jbnu.ac.kr (T.H.); 2 Engineering Materials Center, Korea Institute of Ceramic Engineering and Technology (KICET), Icheon 17303, Republic of Korea; woods3@kicet.re.kr (S.K.); alstn5806@kicet.re.kr (M.-S.N.); 3 Department of Materials Science and Engineering, Korea University, Seoul 02841, Republic of Korea

**Keywords:** reusable TPS, RLV, spacecraft heat shield, HVOF, Cerakwool, TUFI, atmospheric re-entry

## Abstract

We studied a candidate TPS (thermal protection system) material for reusable re-entry space vehicle applications. The material was based on a high-temperature-resistant material called Cerakwool. A total of six specimens were fabricated with substrate densities of 0.45 g/cm^3^, 0.40 g/cm^3^, and 0.35 g/cm^3^, with two specimens for each density. All specimens were coated with high-emissivity TUFI (toughened unpiece fibrous insulation), with coating thicknesses ranging from 445 to 1606 µm. The specimens were tested using an HVOF (high-velocity oxygen fuel) material ablation test facility. For each density specimen pair, one specimen was tested at 1 MW/m^2^ and the remaining one was tested at 0.65 MW/m^2^. The average stagnation point temperature for specimens tested at 1 MW/m^2^ was ~893 °C, approximately 200 °C higher than those tested at 0.65 MW/m^2^. This suggests a ~200 °C increase in stagnation point temperature for a 0.35 MW/m^2^ rise in incident heat flux. During the tests, internal temperatures were measured at three locations. For all tested specimens, regardless of heat flux test conditions and density, the temperature at ~40 mm from each specimen’s stagnation point remained around or below 50 °C, well within the 180 °C design limit set for the TPS back face temperature. Post-test visual inspections revealed no signs of ablation or internal damage, confirming the material’s reusability.

## 1. Introduction

The ready availability and unparalleled widespread use of space technology are on the horizon due to rapid commercialization of the space industry, driven by the entry of major private space organizations like SpaceX and Blue Origin, as well as renewed and accelerated interest from traditional space organizations like NASA.

One significant aspect that will propel this transformation will be the widespread availability of more affordable space launch solutions. RLV (reusable launch vehicle) technology is set to play a pivotal role in the future of the space industry, offering a less expensive space launch capability compared to its counterparts, thanks to its multiple reusability.

Apart from providing economic launch solutions for both payloads and crew, RLVs will address the ever-growing need for innovative technologies capable of retrieving defective satellites from Earth’s orbit for servicing, recycling, or safe disposal, and for swiftly relaunching satellites after servicing. This will promote sustainability in the space industry by conserving valuable hardware and precious rare-earth metals used in sophisticated satellites. This helps to reduce space debris and minimize potential atmospheric pollution caused by the de-orbiting and atmospheric burn-up of defective satellites. Similarly, RLVs can serve as excellent platforms for conducting cutting-edge automated experiments, efficiently returning samples back to Earth, and swiftly relaunching them to space if needed, which is particularly helpful to biopharma research. Though these functions can be served by non-reusable space launch vehicles and non-reusable re-entry spacecraft, the reusability of RLVs provides them with an undeniable competitive edge. RLVs will also aid in the growth of a safe, reliable, robust, and affordable space tourism industry. RLV technology has an indisputable importance in shaping the future of space exploration and its further commercialization. Therefore, the development and maturation of RLV technology are pivotal for maximizing the utilization of space in the future and fostering sustainable space exploration and commercialization.

The categorization of re-entry space vehicles as reusable or non-reusable depends on the classification of the TPS (thermal protection system) materials. Based on their behavior during atmospheric re-entry, TPS materials fall into two categories: 1. reusable, non-ablative, and non-charring; and 2. non-reusable and ablative, which includes both charring and non-charring subtypes [1].

During re-entry, reusable TPS materials remain unchanged both physically and chemically, maintaining their properties before and after the re-entry process. Generally, these materials are designed to withstand the low heat flux conditions encountered during re-entries from low Earth orbit. Reusable TPSs typically re-radiate a significant portion of the heat load while absorbing the remainder; hence, reusable a TPS is sometimes referred to as a ‘heat-soak TPS’. On the other hand, non-reusable TPS materials undergo physical damage and chemical changes during the re-entry process.

The extensive research conducted for the NASA space shuttle program provides robust and invaluable technical insights for RLV development, including reusable TPSs, their constituent materials, and other subsystems. Table 1 shows the first and corresponding second-generation reusable TPS materials developed during the NASA space shuttle program.

Table 2 shows the maximum operating temperatures of the space shuttle first-generation TPS materials/components.

The objective of the current study is to develop and investigate a reusable TPS material for a conceptual winged RLV named ‘JBNU winged RLV’ to perform a similar function as LI tiles or AETB tiles. In this study, we carried out experimental investigations of the reusable TPS candidate material using JBNU’s HVOF material ablation test facility. This facility has been adapted for material testing from its original intended use of powder coating [4] and serves as a preliminary and cost-effective platform for testing space vehicle TPS materials in comparison to plasma wind tunnels (PWTs).

PWTs are traditionally used to test and develop TPS materials, including both ablative and reusable types. Globally, there are two types of PWTs that are commonly used, classified based on how they generate plasma test flow: arc-jet and ICP (inductively coupled plasma) PWTs. In an arc-jet PWT, an inert gas stream (typically argon) is injected into the plasma-generating torch. After the inert gas stream stabilizes, a huge DC voltage is applied across the electrodes. This huge potential difference generates an electric arc within the inert gas stream between the torch’s anode and cathode. A working gas, selected according to the target planet’s atmosphere (for example, air for Earth and CO_2_ for Mars), is then supplied to the torch. The electric arc formed between the electrodes heats the working gas. The intense heat transfer from the arc leads to the dissociation and ionization of the working gas, thereby generating a plasma flow to which the specimens are exposed during tests. In an ICP plasma wind tunnel, the working gas is introduced into a quartz tube that is encircled by a coil. A high-voltage, high-frequency current is applied to the coil, which induces an electromagnetic field inside the quartz tube, creating a solenoid effect. Before introducing the working gas into the quartz tube, a stream of argon is first injected. The strong electric field generated by the coil produces free electrons in the argon stream. These free electrons are then accelerated by the electromagnetic fields, generating eddy currents within the quartz tube. After the working gas is introduced into the quartz tube, the eddy currents generate Joule heating within the gas. This heating causes the working gas to undergo dissociation and ionization, ultimately resulting in the creation of high-enthalpy plasma test flow. Arc-jet PWTs exhibit higher energy deposition rates compared to ICP PWTs; however, the flow in ICP PWTs is contamination-free, as there is no interaction between the metallic parts of ICP PWT torches and the working gas. In contrast, in arc-jets, the working gas interacts directly with the electrodes and other metallic parts of the plasma-generating torch, increasing the possibility of the plasma flow being contaminated by eroded electrodes. Both of these traditional equipment types are expensive to install, operate, and maintain; however, these costs can be justified for advanced testing. For small-scale preliminary investigations, less expensive combustion-based flow-generating equipment, such as oxy-acetylene torches and HVOF torches, are considered. Compared to oxy-acetylene torches, HVOF torches are well developed and studied, as they are widely used in the coating industry. Hence, JBNU has adapted an HVOF torch for TPS material testing.

For a NASA space shuttle, the RCC (reinforced carbon–carbon) was used in the nose cap and wing leading edges as these locations experienced the highest thermal loads during re-entry. HRSI (high-temperature reusable surface insulation) was predominantly used on the bottom surface, i.e., the windward side during re-entry, and also on the vertical tail and rudder leading edges. LRSI (low-temperature reusable surface insulation), AFRSI (advanced flexible reusable surface insulation), and FRSI (flexible reusable surface insulation) were used on the shuttle’s top surface, i.e., the leeward side during re-entry, depending on shuttle local surface temperature during re-entry. Detailed illustrations of TPS material/component distribution on a NASA space shuttle can be found here: [3,5]. AETB (alumina-enhanced thermal barrier) tiles [6], one of the NASA space shuttle program’s second-generation reusable TPS materials, are utilized for current US winged RLVs: Boeing’s X-37B [7] and Sierra Space’s Dream Chaser [8].

NASA also has developed a lightweight reusable TPS composite called TUFROC (toughened uni-piece fibrous reinforced oxidation-resistant composite). TUFROC [9] is a two-piece system, with a fibrous insulation base such as AETB, capped with ROCCI (refractory oxidative-resistant ceramic carbon insulation) [10] surface-treated with HETC (high-efficiency tantalum-based composite) [11]. TUFROC serves as an upgrade to RCC and ACC (advanced carbon–carbon) as the nose cap and wing leading edge TPS material, and have been studied for the X-37B [12] and the Dream Chaser [13].

In Europe, some of the notable reusable re-entry vehicles being developed include SUSIE, Space Rider, and SpaceLiner. SUSIE, which stands for Smart Upper Stage for Innovative Exploration, is a lifting-body re-entry crewed space vehicle being developed by Arianespace, France [14]. Space Rider is also a lifting-body re-entry vehicle designed to function as an uncrewed robotic laboratory. Space Rider stands for Space Reusable Integrated Demonstrator for Europe Return and is developed for ESA primarily by CIRA, Italy. The main TPS material for Space Rider is a CMC (ceramic matrix composite) specifically ISiComp^®^ (a proprietary C/SiC) [15,16]. SpaceLiner is a dual-purpose concept, with hypersonic transportation as its primary role and orbital payload delivery as its secondary role, being developed by DLR, Germany; its TPS will include both NASA shuttle TPS materials and CMCs [17,18,19].

**Figure 1:**
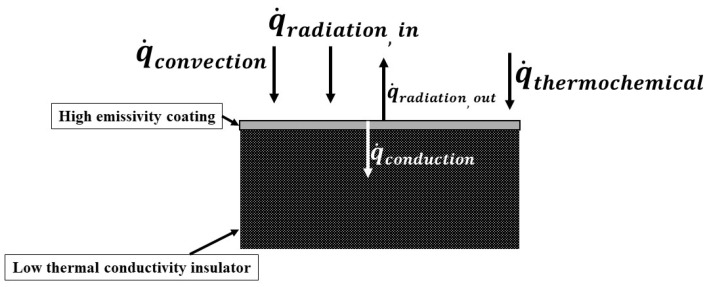
A typical reusable TPS tile material’s characteristics during re-entry.

Figure 1 shows the characteristics of a typical reusable TPS tile material during re-entry.

In typical reusable TPS tiles like LI tiles and AETB tiles, their lower thermal conductivity ensures reduced mass. The surface coatings, such as RCG (reaction cured glass) and TUFI (toughened unpiece fibrous insulation), have high emissivity, which increases the amount of thermal energy re-radiated from the coated TPS tiles. Thus, in typical coated reusable TPS tiles, the incident thermal loads during re-entry are managed by re-radiation and conduction. In contrast, in typical phenolic-based non-reusable ablative TPS materials like PICA (phenolic-impregnated carbon ablator) and the carbon-phenolic/silica-phenolic dual-layer ablator developed by us earlier [20,21,22], pyrolysis and expulsion of mass (i.e., ablation) play significant roles in thermal load management.

During re-entry, the TPS materials must keep their back face temperature below 200 °C, as common high-temperature-resistant aluminum alloys, which are typically used in the construction of re-entry space vehicle metallic frames, start to lose their strength at temperatures above 200 °C [23]. This applies to both reusable and non-reusable TPS materials and their respective re-entry space vehicles. For the JBNU winged RLV (JBNU-Jeonbuk National University), a nominal value of 180 °C was set as the design limit for the reusable TPS back face temperature; for reference, the NASA space shuttles’ aluminum structure design limit temperature was around 177 °C (i.e., 350 °F) [24,25]. The same design limit of 180 °C was set and met by our earlier-developed carbon-phenolic/silica-phenolic dual-layer ablator, where specimens were subjected to a maximum heat flux of 9.4 MW/m^2^ [22].

**Figure 2:**
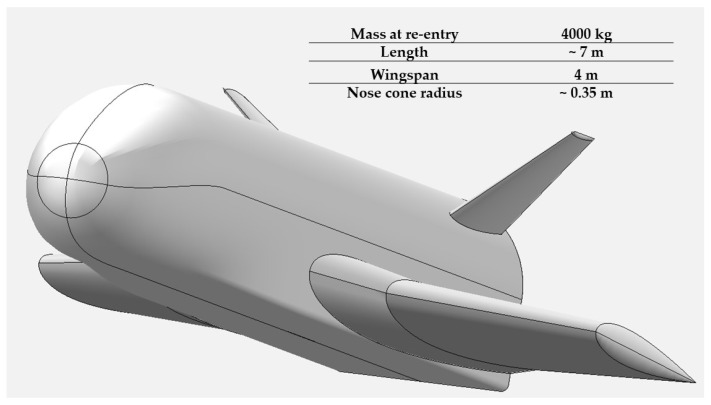
JBNU winged RLV conceptual design.

Figure 2 shows the conceptual design of the JBNU winged RLV.

**Figure 3:**
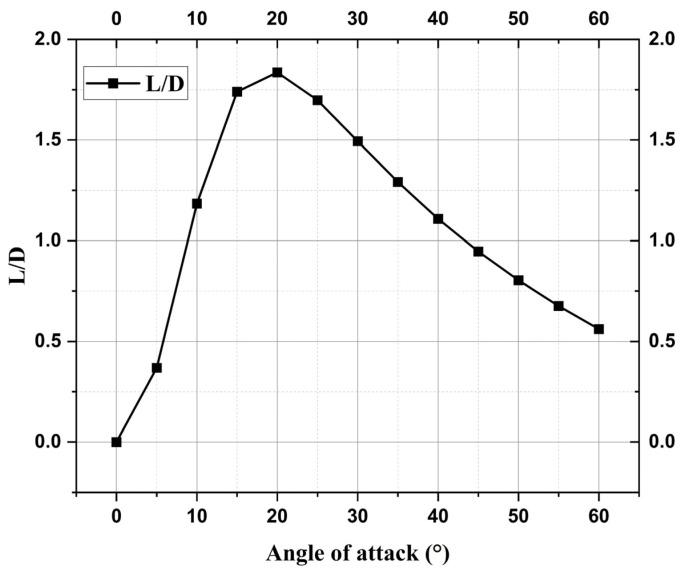
JBNU winged RLV L/D vs. angle of attack.

Using the winged RLV’s geometry profile, hypersonic aerodynamic characteristics can be determined for different angles of attack during re-entry using an analytical method based on Newtonian flow theory [26]. Figure 3 shows the values of L/D (lift-to-drag ratio) for the JBNU winged RLV estimated using the method given in [26].

**Figure 4:**
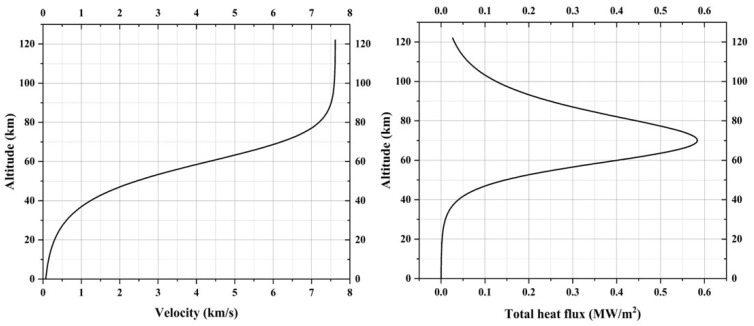
JBNU winged RLV velocity and total heat flux re-entry trajectory profile.

Assuming the JBNU RLV re-enters the atmosphere at a higher angle of attack of 40° with a corresponding L/D ratio of approximately 1, Figure 4 shows the velocity trajectory of the winged RLV for an equilibrium glide path [27]. For a lifting re-entry, high angles of attack and high L/D ratios are desired. High angles of attack minimize the incident thermal load but reduce the winged RLV’s cross range [28]. Higher L/D ratios maximize the cross range [29]; hence, an optimal angle of attack needs to be selected while also considering the corresponding L/D ratio. Figure 4 also shows the JBNU winged RLV nose cap stagnation point heat flux based on the velocity trajectory, calculated as the summation of convective heat flux [30] and radiative heat flux [31].

The values in Figure 4 help determine the conditions for the testing, validation, and development of the reusable RLV and its related components, especially the peak heat flux value for ground testing of reusable TPS candidate materials. The peak heat flux for the JBNU winged RLV is estimated to be around 0.6 MW/m^2^. For reference, the peak heat flux values for NASA space shuttles and the HL-20 (an earlier version of the Dream Chaser) were estimated to be around 0.45 MW/m^2^ [32] and 0.73 MW/m^2^ [33], respectively.

## 2. Materials and Methods

### 2.1. Specimens

The specimens used in this experimental study were fabricated using a high-temperature-resistant material ‘Cerakwool’ [34] as the main material for the base substrate and were finished with TUFI coating [35].

**Figure 5:**
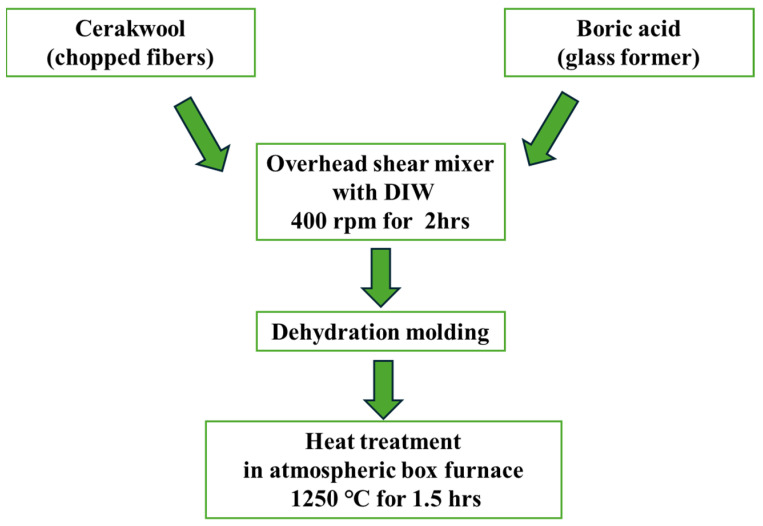
Cerakwool-based substrate fabrication process.

First, Cerakwool in its chopped fiber form was mixed with boric acid, which acts as a glass former, along with deionized water (DIW) using an overhead shear mixer at 400 rpm for 2 h. The mixture was then placed in a casting tower and dehydrated, followed by heat treatment at 1250 °C in an atmospheric box furnace for 1.5 h. Figure 5 illustrates the Cerakwool-based substrate fabrication process. By adjusting the ratios of raw materials, substrates with three different densities of 0.30, 0.40, and 0.45 g/cm^3^ were fabricated. Substrates were fabricated in a cylindrical shape with a diameter of 60 mm and a thickness of 50 mm.

**Figure 6:**
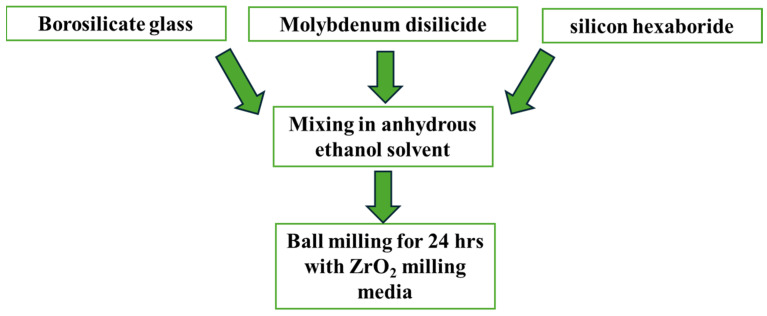
TUFI fabrication process.

**Figure 7:**
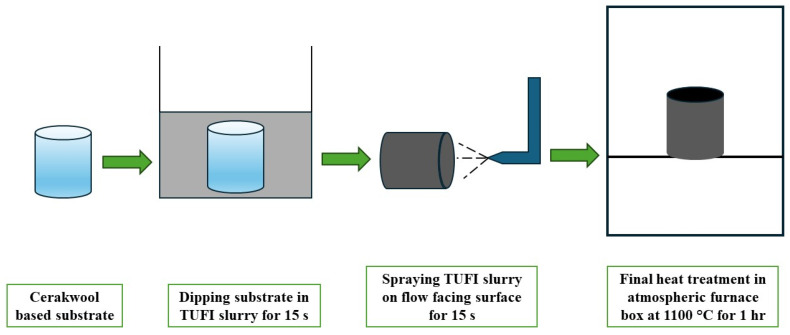
Specimen coating process.

For TUFI fabrication, borosilicate glass, molybdenum disilicide, and silicon hexaboride were mixed with ethanol and then ball-milled with ZrO_2_ grinding media for 24 h. In the resultant slurry, the Cerakwool-based substrates were dipped for 15 s to penetrate the coating mixture into substrates, followed by spraying the TUFI slurry on each substrate’s front surface (i.e., the surface that would be exposed to the test flow) for another 15 s at 0.5 MPa air pressure. As the final step, the coated substrates were heat-treated in an atmospheric box furnace at 1100 °C for 1 h, resulting in the test specimens. Figure 6 illustrates the TUFI fabrication process, and Figure 7 illustrates the TUFI coating process.

**Figure 8:**
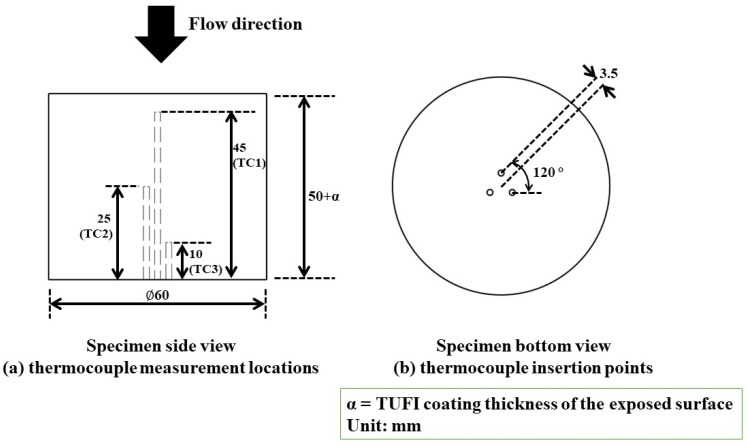
Specimen dimensions showing (**a**) thermocouple measurement locations, (**b**) thermocouple insertion points.

Figure 8 shows the specimen dimensions. Prior to the HVOF tests, each specimen was internally fitted with three K-type thermocouples (accuracy: ±0.75%) to measure internal temperatures when exposed to the test flow. The thermocouples were placed at three locations, 45 mm (TC1), 25 mm (TC2), and 10 mm (TC3), from the back face of the specimen. The thermocouples used were each 1.57 mm in diameter, with insertion points 3.5 mm from the specimen center and positioned at an angle of 120° relative to each other.

**Figure 9:**
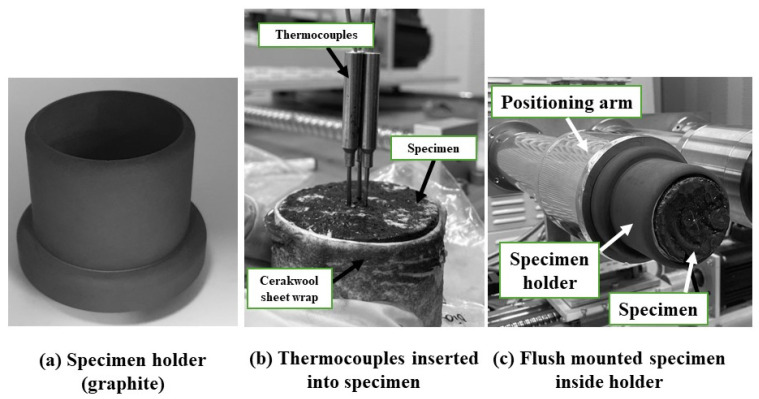
Photographs showing (**a**) the specimen holder, (**b**) thermocouples inserted into a specimen, and (**c**) a specimen mounted in the holder before testing.

To reduce any lateral heat transfer to the specimens during the tests, each specimen was wrapped in Cerakwool fabric sheet pieces and flush-mounted in a graphite specimen holder. Figure 9 shows photographs of the specimen holder, thermocouples inserted into a specimen, and a specimen flush-mounted into the holder prior to a test.

### 2.2. Experimental Setup

**Figure 10:**
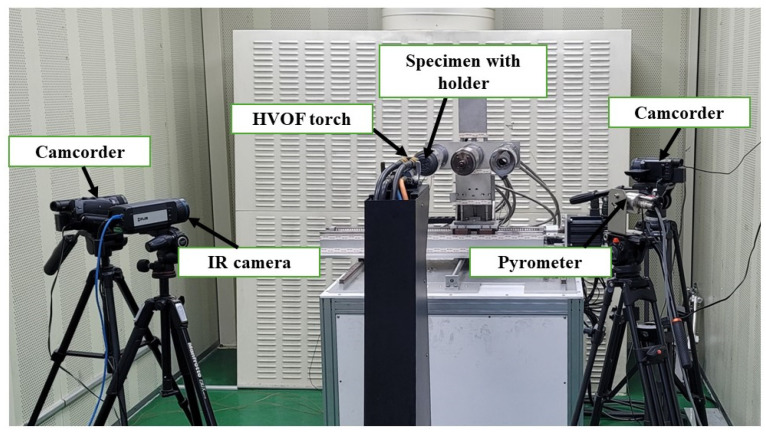
Experimental setup photograph.

Figure 10 shows a photograph of the HVOF test experimental setup. The HVOF torch nozzle has a flow exit diameter of 10.84 mm. Before the material tests, a water-cooled Gardon gauge was used to measure the heat flux in the flow axial direction under a set of operating conditions. The same set of operating conditions was used for the material testing. More details about the HVOF material ablation test facility and Gardon gauge heat flux measurement can be found here: [4,36].

The experimental setup included a two-color pyrometer (IMPAC series ISQ 5 model from LumaSense Technologies, Santa Clara, CA, USA) with a range from 800 °C to 2800 °C (accuracy: ±0.5% up to 1500 °C and ±1% above 1500 °C), and an IR camera (A655SC model from FLIR, Oregon, OR, USA) which can measure temperatures up to 2000 °C (accuracy: ±2%). Both optical devices were used to measure the stagnation point temperature of each specimen during the tests. For the IR camera, an emissivity value of 0.873 (i.e., the emissivity of TUFI [37]) was used.

A total of 6 specimens were tested. Two specimens were fabricated per density of the base substrate; one was tested at 0.65 MW/m^2^, which is closer to the peak heat flux value estimated for JBNU RLV re-entry (see Figure 4), and the other was tested at 1 MW/m^2^. Under both heat flux test conditions, specimens were tested for 30 s. During the tests, data from three internal thermocouples were acquired using a National Instruments NI cRIO-9054 (a real-time embedded industrial controller) with an NI 9212 thermocouple input module. After the experiments, a Smartzoom 5 digital microscope from ZEISS, Oberkochen, Germany, was used to inspect cross-sections and visually measure the thickness of the TUFI coating of the tested specimens. Table 3 shows the specimen test conditions and the TUFI coating thickness for each specimen.

## 3. Results and Discussion

**Figure 11:**
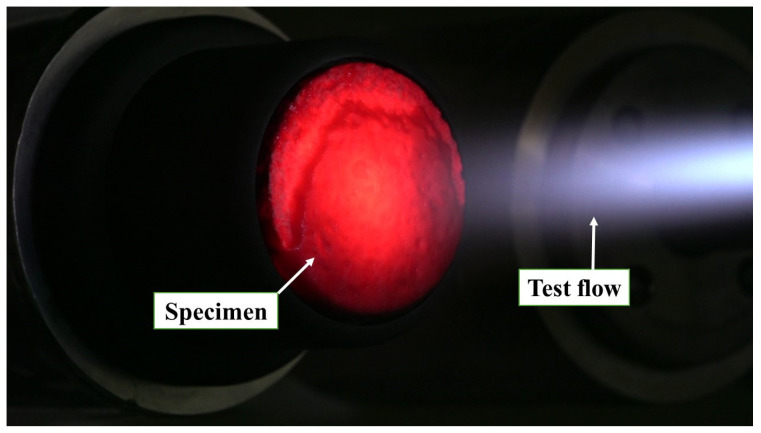
Photograph of a specimen being exposed to the test flow (A2, 1 MW/m^2^).

Figure 11 shows a photograph of a specimen being exposed to the test flow during the experiment. In the initial tests, the stagnation point temperatures of the specimens were below 800 °C, which is the lower limit measurable by the pyrometer. Consequently, the pyrometer was not used for the remaining tests.

**Figure 12:**
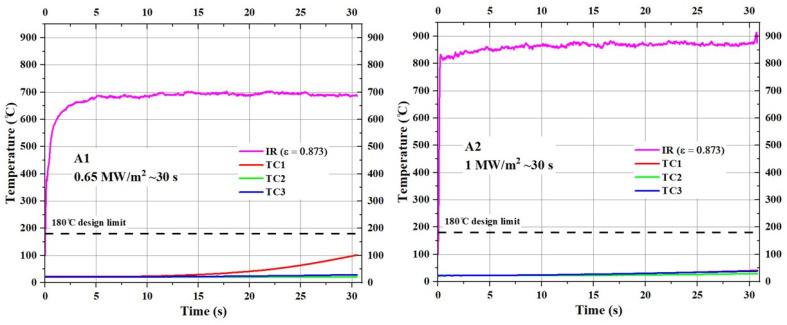
Temperature responses of type A specimens (A1 and A2).

Figure 12 shows the temperature responses of type A specimens (substrate density = 0.45 g/cm^3^) during the tests.

For the A1 specimen, tested at 0.65 MW/m^2^, the trend shown in Figure 12 indicates that the stagnation point temperature stabilized around 700 °C during the test. In contrast, for the A2 specimen, tested at 1 MW/m^2^, the stagnation temperature stabilized between 850 °C and 900 °C, representing an increase of approximately 150 °C to 200 °C in surface temperature for a 0.35 MW/m^2^ increase in incident heat flux. For both specimens, the internal temperatures at all measured locations were well below the set design limit of 180 °C, with all temperatures remaining below 50 °C, except for the TC1 temperature of the A1 specimen, which increased to around 100 °C by the end of the test duration.

**Figure 13:**
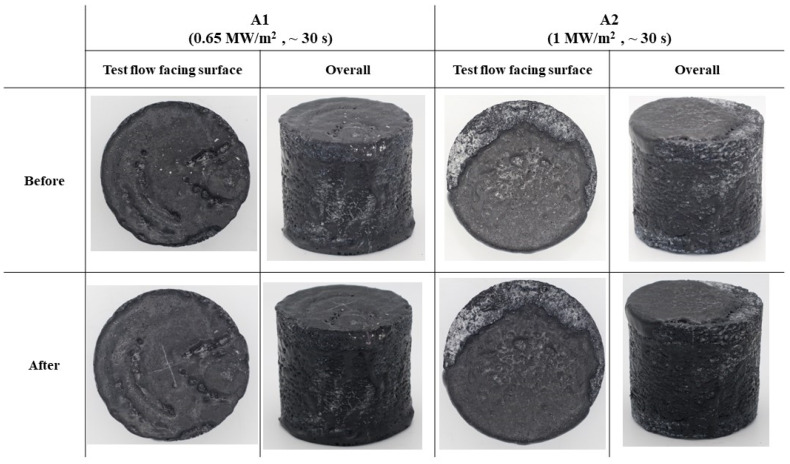
Type A specimens (substrate density = 0.45 g/cm^3^) before and after test photographs.

Figure 13 shows before and after test photographs of the type A specimens. The visual inspection of these specimens revealed no signs of ablation, which is a major factor in determining the reusability of TPS materials.

**Figure 14:**
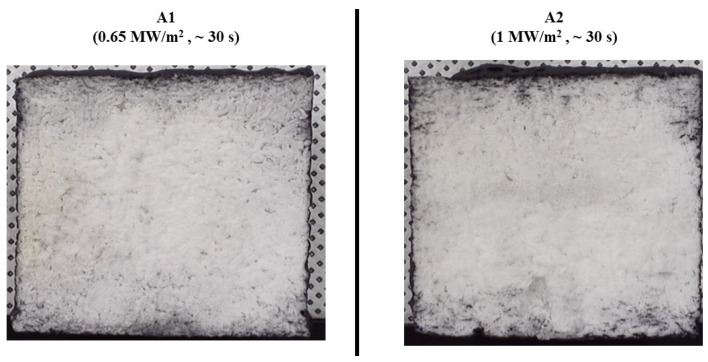
Type A specimens (substrate density = 0.45 g/cm^3^) after test cross-section images.

Figure 14 shows images of the cross-sections of the type A specimens after the tests. The photographs reveal no signs of internal damage, confirming the reusability of the tested specimens.

**Figure 15:**
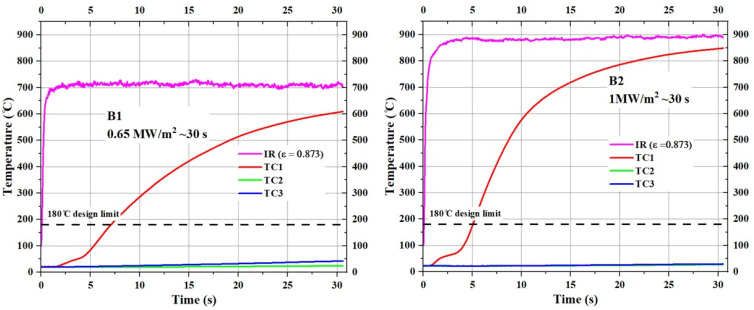
Temperature responses of type B specimens (B1 and B2).

Figure 15 shows the temperature responses of type B specimens (substrate density = 0.40 g/cm^3^) during the tests.

The stagnation point temperature of the B1 specimen stabilized between 700 °C and 750 °C, whereas the stagnation point temperature of the B2 specimen stabilized slightly below 900 °C. Although the trends in TC2 and TC3 temperatures for type B specimens, as shown in Figure 15, are similar to those of the TC2 and TC3 temperatures of their type A counterparts, the TC1 temperatures of the type B specimens increased very close to their respective stagnation point temperatures.

**Figure 16:**
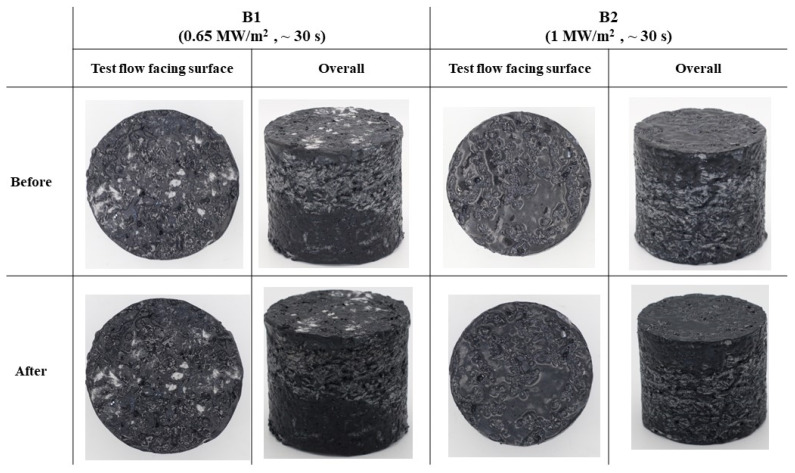
Type B specimens (substrate density = 0.40 g/cm^3^) before and after test photographs.

**Figure 17:**
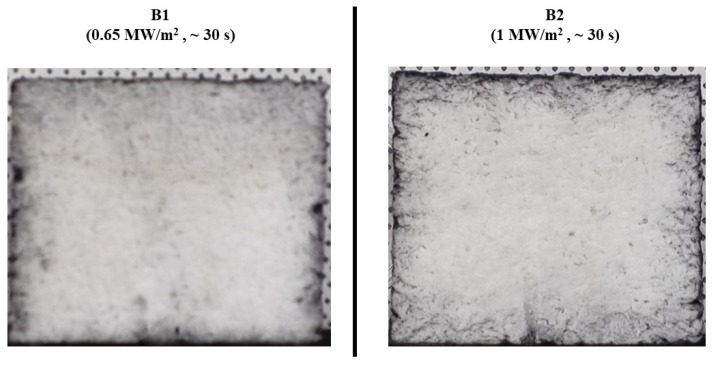
Type B specimens (substrate density = 0.40 g/cm^3^) after test cross-section images.

Figure 16 shows before and after test photographs of the type B specimens, and Figure 17 displays images of the cross-sections of the type B specimens after the tests. Both figures indicate no visual signs of surface ablation or internal damage in the type B specimens.

**Figure 18:**
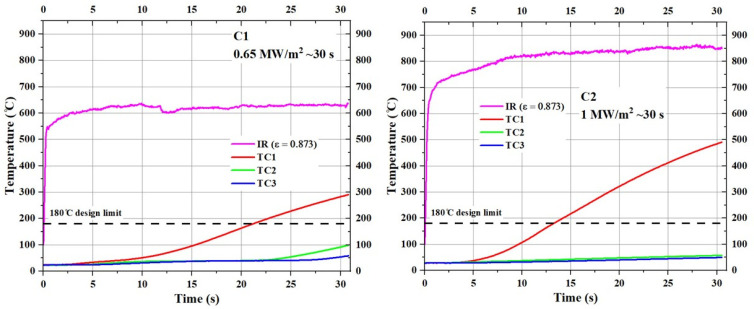
Temperature responses of type C specimens (C1 and C2).

Figure 18 shows the temperature responses of the type C specimens (substrate density = 0.35 g/cm^3^) during the tests.

The stagnation point temperature of the C1 specimen stabilized between 600 °C and 650 °C, whereas the stagnation point temperature of the C2 specimen stabilized around 850 °C. The maximum stagnation point temperatures for the A1, B1, and C1 specimens (tested at 0.65 MW/m^2^ for ~30 s) were 703.80 °C, 730.30 °C, and 638.39 °C, respectively. In contrast, the maximum stagnation point temperatures for the A2, B2, and C2 specimens (tested at 1 MW/m^2^ for ~30 s) were 914.20 °C, 900.57 °C, and 864.74 °C, respectively, indicating an average increase of ~200 °C for a 0.35 MW/m^2^ increase in incident heat flux. At the end of their respective tests, the TC1 temperatures of the C1 and C2 specimens increased to ~300 °C and ~500 °C, respectively. At the end of the test, the TC2 temperature of the C1 specimen increased by ~100 °C and the TC3 temperature increased by ~50 °C, whereas, for the C2 specimen, both TC2 and TC3 temperatures at the end of the test were around 50 °C.

**Figure 19:**
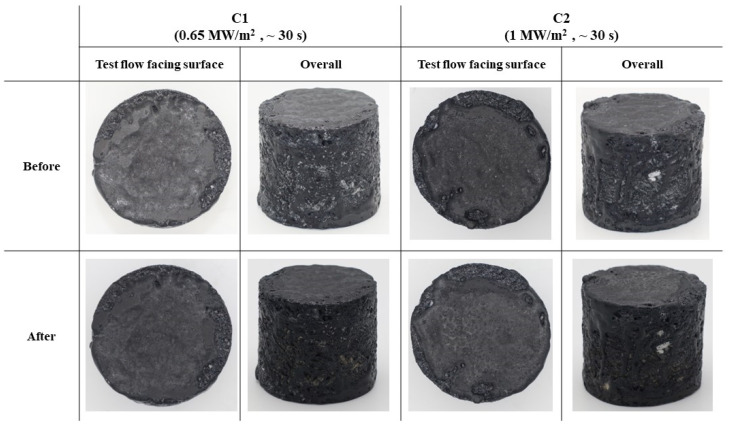
Type C specimens (substrate density = 0.35 g/cm^3^) before and after test photographs.

**Figure 20:**
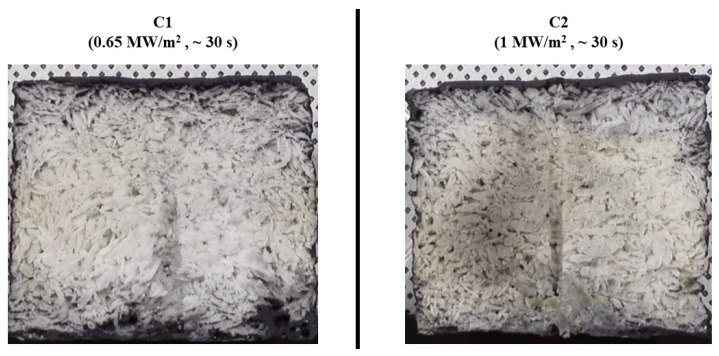
Type C specimens (substrate density = 0.40 g/cm^3^) after test cross-section images.

Figure 19 shows before and after test photographs of the type C specimens, and Figure 20 displays images of the cross-sections of the type C specimens after the test. As seen in both type A and B specimens, the type C specimens also showed no signs of surface ablation or internal damage.

**Figure 21:**
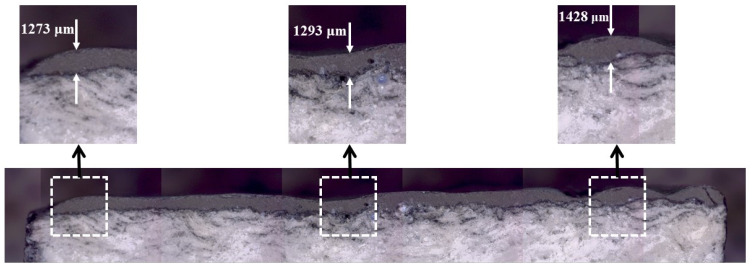
A1 specimen post-test flow-exposed surface cross-section magnified image.

Figure 21 shows a magnified cross-section image of the flow-exposed surface of the A1 specimen post-test. In Figure 21, the TUFI coating and Cerakwool-based substrate are visible as distinct layers, with the TUFI coating appearing as a darker shade of gray and the Cerakwool-based substrate as a lighter shade of gray. From Figure 21, it can also be observed that the base substrate is more porous than the coating layer. For representative purposes, only three thickness measurement points are shown in Figure 21. However, the estimated average thickness value for the A1 specimen was based on many more measurement points than those displayed in Figure 21.

The mass of the tested specimens was measured before and after the tests (see Table 4). Interestingly, a slight mass increase was observed in the A1, A2, C1, and C2 specimens. In contrast, the B1 and B2 specimens showed substantial mass losses. Since no surface ablation was observed in the B1 and B2 specimens, the mass losses are believed to have occurred due to mechanical abrasion during handling, such as the insertion and removal of the thermocouples before and after the tests. This is attributed to the Cerakwool-based substrate being brittle and possibly crumbled if not handled properly, which resulted in mass loss during the installation and removal of the thermocouples in the B1 and B2 specimens. Additionally, the slight mass increases in the A1, A2, C1, and C2 specimens are attributed to the adhesive used to secure the thermocouples before the tests, which remained as residue after the removal of the thermocouples at the end of the tests.

In all tested specimens, TC3 temperatures, the farthest measurement location from the specimen stagnation points, were around and below 50 °C, a value well below the set design limit of 180 °C; thus, this satisfies one of important selection criteria along with the lack of ablation and internal damage due to the tests. The results obtained from the specimens are considered satisfactory overall. However, further advanced investigations are needed to confirm these results and to gain a deeper understanding of the material’s behavior under such test conditions.

Since there is not much overall performance difference among the specimens of different densities tested in this study, specimens with densities lower than those studied will be fabricated and investigated. In this study, the TUFI coating thickness was not standardized among the specimens, resulting in varying levels of coating thickness. Future studies will aim to standardize the TUFI coating process to achieve uniformity in coating thickness across all test specimens. In the future, specimens with different levels of TUFI coating and/or new high-emissivity coatings will also be tested. Additionally, materials like aluminoborosilicate or alumina, which are used in NASA tiles, will be introduced in the specimen fabrication process to enhance the overall performance of the studied reusable TPS materials.

Future plans also include various experiments for optimizing the overall synthesis process. The parameters that will be modified for these experiments include the temperature and pressure maintained during the material fabrication process, along with the previously mentioned changes in material compositions and improvements in coating techniques. By systematically studying how these parameters affect the material’s thermal insulation, mass, and overall strength, they can be refined to achieve both an optimal material and synthesis parameters for the reusable TPS materials.

Future work will also focus on studying the impact of impurities on the thermal insulation properties of reusable TPS materials. Using the materials tested in this study as a baseline, the level of impurities will be assessed, and the effects of these impurities on material properties will be studied in detail. For example, the presence of impurities in silica reusable TPS tiles (i.e., LI tiles) causes crystallization and has a major effect on fiber shrinkage [38,39]. Hence, for manufacturing silica tiles, it is recommended to take great care to avoid contamination by using high-quality raw materials, washing fibers with dilute hydrochloric acid, rinsing with deionized water, and utilizing stainless steel or polyethylene utensils while agitating slurries with pure nitrogen [40]; the ideal impurity content in silica tiles should be less than 0.3% and the total alkali and alkaline earth content should be kept below 0.06% [38]. So, careful analysis of raw materials to detect the presence of any impurities is required, along with thorough scrutiny of manufacturing equipment and techniques to prevent contamination during the fabrication process. Studies have shown the presence of sodium-based and lithium-based impurities in TUFROC [41], as well as impurities like calcium, sodium, and magnesium observed in SiSiC-based heteroporous heterogeneous ceramic reusable TPS material [42]; however, how these impurities affect the overall TPS performance is not very clear.

Some impurities can be beneficial and help improve thermal insulation, while others may be detrimental and reduce thermal insulation; the same can be said for other material properties.. For example, in some UHTC TPS materials, metallic impurities aid in the densification process, while oxide impurities, though not beneficial for densification, are helpful for grain growth [43]. Apart from inheriting impurities from raw materials and cross-contamination from manufacturing equipment, improper, incomplete, and incorrect fabrication procedures also lead to undesirable compounds, i.e., impurities, in the final product, such as the presence of unreacted raw materials. For example, in the case of SiC-based TPS, if SiC is manufactured at lower temperatures, it tends to have free silicon atoms, which act as impurities [44]. In some TPS materials, undesirable porosity or improper pore distribution can also be considered impurities, as they affect the performance of the TPS material. Environmental contamination can also occur when fabricated TPS materials absorb impurities from the environment, which can hinder the performance of the TPS. For example, silica-based reusable TPS materials are known to absorb moisture from the environment, which can lead to an increase in mass, expansion of the material due to the turning of moisture to ice at higher altitudes, and/or instant destruction of the material due to the rapid boiling of moisture during re-entry. Hence, proper treatment of TPS materials is needed after fabrication [45].

So, it becomes very important to assess the level of impurities and their impact at each stage of the TPS material fabrication process, as well as post fabrication; this will be addressed in detail in future studies.

## 4. Conclusions

Candidate material specimens for reusable TPS applications were fabricated and investigated using a combustion-fuel-based material ablation test facility. The substrates for the specimens were made from a high-temperature-resistant material called Cerakwool. Three different density substrates were fabricated: 0.45 g/cm^3^, 0.40 g/cm^3^, and 0.35 g/cm^3^. Each substrate was coated with a high-emissivity TUFI coating. In total, six specimens were fabricated, with two specimens for each substrate density. TUFI coating thickness ranged from 445 to 1606 µm.

In each density specimen pair, one specimen was tested at ~0.65 MW/m^2^ and the other was tested at ~1 MW/m^2^. These heat flux test conditions were selected based on the re-entry heat flux trajectory of a conceptual winged RLV named the ‘JBNU Winged RLV’. Each test had a duration of ~30 s.

The average stagnation point temperature of the specimens tested at ~1 MW/m^2^ is ~893 °C, which is around 200 °C higher than the average stagnation point temperature of the specimens tested at ~0.65 MW/m^2^. This indicates an approximate increase of ~200 °C in stagnation point temperature for a 0.35 MW/m^2^ increase in incident heat flux.

During the tests, the internal temperatures measured at ~5 mm from each specimen’s stagnation point varied significantly among the tested specimens. However, the internal temperatures measured at ~25 mm (except for one specimen) and ~40 mm remained around or below 50 °C, thus satisfying the design criteria limit of 180 °C.

Visual inspections of the post-test images of each specimen’s surface and cross-section revealed no signs of ablation or internal damage, indicating the reusability of the specimens.

Four specimens showed a slight increase in mass after the tests, which is attributed to the residue of adhesive used for securing the thermocouples inside the specimens. In contrast, the remaining two specimens experienced a mass reduction, which is attributed to physical damage that may have occurred during the handling of those two specimens.

In a nutshell, the overall results are satisfactory, but further advanced investigations are needed to confirm them and better understand the material’s behavior under these conditions.

## Figures and Tables

**Table 1:** NASA space shuttle first- and second-generation TPS materials [2].

First-Generation Shuttle TPS	Second-Generation TPS
Reinforced carbon–carbon (coated)	Advanced carbon–carbon
LI tiles (with RCG coating)	AETB tiles (with TUFI coating)
AFRSI (with C-9 coating)	TABI and CFBI (with PCC coating)
FRSI (with DC92 coating)	PBI felt (with C-9 coating)

**Table 2:** Space shuttle TPS maximum operating temperature [3].

Space Shuttle TPS Component	Maximum Operating Temperature (°C)	
	100-Mission Life	Single-Mission Life
RCC	1482	1816
HRSI (LI-900 with black RCG coating)	1260	1427
LRSI (LI-900 with white RCG coating)	1093	1149
AFRSI	816	982
FRSI	399	482

**Table 3:** Specimen test conditions.

No.	Specimen	Density (g/cm^3^)	Heat Flux (MW/m^2^)	^†^ TUFI Coating Thickness on the Exposed Surface (µm)	Distance from the HVOF Nozzle Exit (mm)
1	A1	0.45	0.65	1264 ± 391	260
2	A2		1	1606 ± 769	220
3	B1	0.40	0.65	445 ± 182	260
4	B2		1	825 ± 235	220
5	C1	0.30	0.65	1115 ± 406	260
6	C2		1	1348 ± 298	220

Each test duration = ~30 s; ^†^ measured visually from magnified specimen cross-section images of surface exposed to the test flow.

**Table 4:** Specimen mass before and after test.

Specimen	Before Mass (g)	After Mass (g)	∆mass * (g)
A1	70.86	70.93	0.07
B1	55.59	54.53	−1.06
C1	53.33	53.44	0.11
A2	67.87	68	0.13
B2	67.03	61.73	−5.3
C2	51.51	52.54	1.03

* ∆mass = after mass − before mass.

## Data Availability

The data will be made available on request from the corresponding author.

## Nomenclature

ACC	advanced carbon–carbon
AETB	alumina-enhanced thermal barrier
AFRSI	advanced flexible reusable surface insulation
C_D_	drag coefficient
CFBI	composite flexible blanket insulation
CIRA	Centro Italiano Ricerche Aerospaziali
C_L_	lift coefficient
CMC	ceramic matrix composite
DIW	deionized water
DLR	Deutsches Zentrum für Luft- und Raumfahrt
ESA	European Space Agency
FRSI	flexible reusable surface insulation
HETC	high-efficiency tantalum-based composite
HRSI	high-temperature reusable surface insulation
HVOF	high-velocity oxygen fuel
ICP	inductively couple plasma
JBNU	Jeonbuk National University
L/D	lift-to-drag ratio
LI	Lockheed insulation
LRSI	low-temperature reusable surface insulation
NASA	National Aeronautics and Space Administration
NI	National Instruments
PBI	polybenzimidazole insulation
PCC	protective ceramic coating
PICA	Phenolic-impregnated carbon ablator
PWT	plasma wind tunnel
$\dot{q}$	heat flux
RCC	reinforced carbon–carbon
RCG	reaction cured glass
RLV	reusable launch vehicle
ROCCI	refractory oxidative-resistant ceramic carbon insulation
SPOF	single point of failure
Space Rider	Space Reusable Integrated Demonstrator for Europe Return
SUSIE	Smart Upper Stage for Innovative Exploration
TABI	tailorable advanced blanket insulation
TC	thermocouple
TPS	thermal protection system
TUFI	toughened uni-piece fibrous insulation
TUFROC	toughened uni-piece fibrous reinforced oxidation-resistant composite
TSTO	two-stage-to-orbit
UHTC	ultra-high temperature ceramic
